# Mothers’ HIV status and their children’s nutritional status: Insights from secondary analysis of the Zimbabwe Demographic and Health Survey data (2015–2016)

**DOI:** 10.1002/fsn3.2509

**Published:** 2021-08-07

**Authors:** Godfrey N. Musuka, Tafadzwa Dzinamarira, Diego F Cuadros, Grant Murewanhema, Innocent Chingombe, Felicia Takavarasha, Helena Herrera, Munyaradzi Mapingure

**Affiliations:** ^1^ ICAP at Columbia University Harare Zimbabwe; ^2^ Department of Geography and Geographic Information Science University of Cincinnati Cincinnati OH USA; ^3^ Unit of Obstetrics and Gynaecology Faculty of Medicine and Health Sciences University of Zimbabwe Harare Zimbabwe; ^4^ Independent Consultant Amsterdam Netherlands; ^5^ University of Portsmouth Portsmouth UK

**Keywords:** children, HIV, nutrition, Zimbabwe

## Abstract

Infants and young children are completely dependent on others, primarily their mothers, for nutrition. This means maternal health status is one of the most important maternal characteristics that are predictors of the nutritional status of children. This study aimed to describe the association between mothers’ HIV status and their children's nutritional status using data from the Zimbabwe Demographic and Health Survey (2015–16). We used statistical analysis to determine the association between mothers’ HIV status and their children's nutritional status. The findings revealed that 30%, 4%, and 11% of children whose mothers were HIV positive presented with moderate‐to‐severe stunting, wasting, and underweight, respectively. The risk of stunting was higher for children whose mothers were HIV positive compared with children whose mothers were HIV negative (odds ratio [OR] 1.23; 95% confidence interval [CI] 1.00–1.52)). Maternal HIV‐positive status is associated with an increased risk of the child being underweight (OR 1.69; 95% CI 1.24–2.30). The prevalence of being underweight, stunting, and wasting is still high among children of HIV‐positive mothers several years into HIV Care and Treatment programs. This study's findings call for implementation of a robust national wide improved infant and young child feeding scheme to enhance the overall nutritional status of children in the country.

## Graphical abstract

Infants and young children are completely dependent on others, primarily their mothers, for nutrition. This means maternal health status is one of the most important maternal characteristics that are predictors of the nutritional status of children. In this paper, we conducted to describe the association between mothers’ HIV status and their children's nutritional status using data from the Zimbabwe Demographic and Health Survey (2015–16).

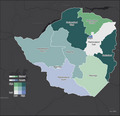

## 1 INTRODUCTION

HIV and childhood malnutrition remain significant public health challenges in sub‐Saharan Africa. As of 2019, Zimbabwe has an HIV prevalence of 14%, which is one of the highest globally. In the 15–49 age‐group, the prevalence is 11.8% (Ministry of Health and Child Care (MoHCC), 2021). Women are disproportionately affected and constitute an estimated 67% of people living with HIV/AIDS (PLWHA) in Zimbabwe. The prevalence of HIV among women is 14.8% compared with 8.6% for their male counterparts.

The World Health Assembly global target for 2030 stipulates the need to “end all forms of malnutrition” (Sustainable Development Goal 2) or achieve a “50% reduction in the number of children under 5 who are stunted” (General Assembly, 2015). However, as of 2019, 21.9%, 7.3%, and 41.7% of children under 5 years old globally were stunted, wasted, and anemic, respectively (Organization, 2019). In Zimbabwe, 23,5% and 2.9% of children under 5 years of age were stunted and wasted, respectively (Global Nutrition Report, 2019). These figures are lower than the average for the Africa region. Zimbabwe is therefore on course to achieve the targets for child nutrition.

Infants and young children are completely dependent on others, primarily their mothers, for nutrition. The United Nations International Children's Emergency Fund (UNICEF) framework on malnutrition highlights “nurturing care” as a critical requirement for healthy child growth and development (Global Nutrition Report, 2019; Organization, 2019). This means maternal health status is one of the most important maternal characteristics that are predictors of the nutritional status of children. This study aimed to describe the association between mothers’ HIV status and their children's nutritional status using data from the Zimbabwe Demographic and Health Survey (ZDHS) conducted in 2015.

## 2 MATERIALS AND METHODS

### 2.1 Study area and data sources

The Demographic and Health Surveys (DHS) Program has collected, analyzed, and disseminated accurate and representative data on population, health, HIV, and nutrition through more than 400 surveys in over 90 countries. The survey is conducted every five years. In Zimbabwe, the latest survey data were collected in the last quarter of 2015 and first quarter of 2016, hence the notation 2015–2016 and this was chosen for this analysis as it provides the most recent data available. The ZDHS methodology has been described elsewhere (National Statistics Agency (ZIMSTAT), 2015). Briefly, subjects were enrolled in the ZDHS via a two‐stage sampling procedure to select households. A total of 400 ZDHS sample locations were selected. The study population was limited to 5,575 mothers who had a definitive HIV status and had at least one of her children 0 to 59 months old surveyed. Anonymous HIV testing was performed on the mothers, with the informed consent of all sampled individuals. HIV serostatus was determined by testing with the enzyme‐linked immunosorbent assay (ELISA) Vironostika Uniform 2 Ag/AB. All samples that tested positive and a random sample of 10% of samples that tested negative were retested with a second ELISA, the Enzygnost^®^ HIV Integral II assay (Siemens). Positive samples on both tests were classified as HIV positive. If the first and second tests were discordant, the two ELISAs were repeated; if the results remained discordant, a confirmatory test, the HIV 2.2 Western blot (DiaSorin), was administered. Procedures and questionnaires for standard Demographic Health Surveys (DHS) have been reviewed and approved by the ICF International Institutional Review Board (IRB). Additionally, country‐specific DHS survey protocols are reviewed by the ICF IRB and typically by an IRB in the host country specifically the Medical Research Council of Zimbabwe.

In the 2015 ZDHS, children under age 5 had their height and weight measured to assess their nutritional status. Weight and recumbent length were measured for children age 0–23 months. Weight and standing height were also measured for children age 24–59 months from the sampled households regardless of whether the mother was interviewed in the survey. Children's height/length, weight, and age data were used to calculate three indices: height‐for‐age, weight‐for‐height, and weight‐for‐age. Each of these indices is expressed in terms of standard deviations from the median (Z‐scores) of the WHO reference population (WHO Child Growth Standards, 2006). Height‐for‐age is a measure of linear growth retardation and cumulative growth deficits. Children whose height‐for‐age Z‐score is below minus two standard deviations (−2 *SD*) from the median of the reference population are considered short for their age (stunted), or chronically undernourished. Children who are below minus three standard deviations (−3 *SD*) are considered severely stunted (National Statistics Agency (ZIMSTAT), 2015). The weight‐for‐height index measures body mass in relation to body height or length and describes current nutritional status (WHO Child Growth Standards, 2006). Children whose Z‐score is below minus two standard deviations (−2 *SD*) from the median of the reference population are considered thin (wasted), or acutely undernourished. Children whose weight‐for‐height Z‐score is below minus three standard deviations (−3 *SD*) from the median of the reference population are considered severely wasted. Weight‐for‐age is a composite index of height‐for‐age and weight‐for‐height. It considers both acute and chronic undernutrition. Children whose weight‐for‐age Z‐score is below minus two standard deviations (−2 *SD*) from the median of the reference population are classified as underweight. Children whose weight‐for‐age Z‐score is below minus three standard deviations (−3 *SD*) from the median are considered severely underweight. Children whose weight‐for‐height Z‐score is more than 2 standard deviations (+2 *SD*) above the median of the reference population are considered overweight. Any anemia in children is defined as a blood hemoglobin level below 11.0 g/dl. In the 2015 ZDHS, severe anemia is defined as <7.0 g/dl, and moderate anemia is defined as 7.0–9.9 g/dl.

Blood specimens were collected for anemia testing from all children age 6–59 months. Hemoglobin analysis was conducted on site with a battery‐operated portable.

HemoCue^®^ analyzer, which produces a result in less than one minute.

### 2.2 Statistical analysis

STATA version 16.1, Texas USA, was used to conduct statistical analysis. We used simple proportion to describe the characteristics of the women and their children included in the analysis. Statistical significance cutoff for purposes of describing the association between maternal HIV status and nutritional status of the children was set at *p* <.05. Odds ratio and their 95% confidence intervals were also used to establish risk factors for stunting, being underweight, and anemia among the children. Outcomes considered for this analysis were stunting, being underweight and anemia. These are binary outcomes and were able to fit univariate logistic regression models for various risk factors such as demographics and maternal HIV status. A multivariate logistic regression model was also fitted in STATA since the nature of exposures and outcomes can follow a logit model

Logit(π(X))=β0+β1X1+…+βpXp

where the probability of the binary outcome, for example being stunted or not can be calculated simultaneously for various risk factors combined and expressed as odds ratios.

We also generated bivariate maps to illustrate the spatial distribution of the different nutritional factors at provincial level. These maps were generated using ArcGIS PRO (Esri, 2020).

## 3 RESULTS

### 3.1 Demographic Characteristics

In this analysis, children's gender was equally distributed being 49% male and 51% female (Table 1). Consistent with the Zimbabwe population, around two thirds of the participants were from rural areas and close to two thirds of the mothers had normal body mass index (BMI = weight/height2) and the same fraction had secondary education. Apostolic sect was the dominant religion at 48%.

**TABLE 1:** Mother and baby demographic characteristics for ZDHS 2015 participants included in this analysis

Variable	Frequency *n* (%)
	*N* max = 5,575
Children age‐group in months	
<6	552 (11)
06	249 (5)
09	243 (5)
17	515 (11)
18–23	479 (10)
24–35	948 (19)
36–47	919 (19)
48–59	893 (19)
Mother age‐group in years	
15–19	366 (6)
20–24	1,389 (24)
25–29	1,489 (28)
30–34	1,262 (23)
35–39	687 (13)
40–44	328 (6)
45–49	54 (1)
Children gender	
Male	2,753 (49)
Female	2,822 (51)
Residence	
Urban	2,062 (32)
Rural	3,513 (68)
Mother's nutritional status	
Thin (BMI <18.5)	239 (4)
Normal (BMI 18.5–24.9)	3,262 (60)
Overweight/obese (BMI ≥25)	2,024 (36)
Education level	
No education	56 (1)
Primary	1,639 (31)
Secondary	3,570 (63)
Tertiary of higher	310 (5)
Religion	
Traditional	29 (1)
Roman catholic	268 (5)
Protestant	729 (13)
Pentecostal	1,323 (22)
Apostolic sect	2,542 (48)
Other Christian	319 (4)
Muslim	16 (0)
None	341 (6)
Other	8 (0)
Wealth quintile	
Poorest	1,149 (23)
Poorer	980 (19)
Middle	889(17)
Richer	1,460 (24)
Richest	1,097 (17)

#### 3.1.1 Stunting

A large proportion of children whose mothers are HIV positive have moderate‐to‐severe stunting compared with those whose mothers are HIV negative, 30% versus 25%, *p* = .008. Although not statistically significant, the same pattern is shown when considering those who are severely stunted, 9% versus 7%, *p* = .201. (Table 2).

**TABLE 2:** Nutritional status of the children by HIV status of the biological mother

Nutritional status of children:	Mother HIV Positive	Mother HIV Negative	*p*‐Value
	*n* (%)	*n* (%)	
Moderate to severe stunting			
No	485 (70)	2,980 (75)	.008
Yes	207 (30)	959 (25)	
Severe stunting			
No	627 (91)	3,651 (93)	.201
Yes	65 (9)	288 (7)	
Moderate to severe wasting			
No	660 (96)	3,770 (96)	.643
Yes	26 (4)	138 (4)	
Severe wasting			
No	677 (99)	3,860 (99)	.564
Yes	9 (1)	48 (1)	
Moderate‐to‐severe underweight			
No	627 (89)	3,692 (93)	.001
Yes	71 (11)	273 (7)	
Severe underweight			
No	685 (98)	3,915 (99)	.059
Yes	13 (2)	50 (1)	
Overweight			
No	645 (94)	3,666 (94)	.866
Yes	41 (6)	242 (6)	
Anemia			
Not anemic	365 (58)	2,147 (62)	.002
Mild	130 (21)	769 (22)	
Moderate	117 (20)	505 (15)	
Severe	8 (1)	13 (0)	

#### 3.1.2 Wasting

The prevalence of moderate‐to‐severe wasting is very low and not significantly different between children whose mothers are HIV positive and those whose mothers are HIV negative, 4% versus 4%, *p* = .0623. This pattern does not change when we restrict to severe wasting. (Table 2).

#### 3.1.3 Underweight

A significantly large proportion of children whose mothers are HIV positive are underweight compared with children whose mothers are HIV negative, 11% versus 7%, *p* = .001. Although it is of borderline statistical significance, the same pattern is shown when we restrict to children who are severely underweight, 2% versus 1%, *p* = .057. (Table 2).

#### 3.1.4 Overweight

There are no differences between overweight proportions between children whose mother are HIV positive and children whose mothers are HIV negative, 6% versus 6%, *p* = .866 (Table 2).

#### 3.1.5 Anemia

A smaller proportion of children whose mothers are HIV positive is not anemic compared with the children born to mothers who are HIV negative (58% versus 62%), moderate anemia proportions are higher among children of HIV positive mothers compared with children of HIV‐negative mothers (20% versus 15%), and so is the case for severe anemia (1% versus 0%), overall *p* = .002. (Table 2).

### 3.2 Univariate and multivariate analyses

#### 3.2.1 Stunting

From the univariate analysis with reference to stunting, in the first 5 years of life stunting differed by gender with females less likely to be stunted as compared to males, unadjusted odds ratio (95%CI) 0.77 (0.66–0.90). Stunting differed by area of residence with the children residing in rural areas being more likely to be stunted, unadjusted odds ratio (95% CI) 1.35 (1.13–1.61). The risk of stunting was highest for children whose mothers were thin, when compared to those with normal BMI or those who are obese, OR (95% CI) 0.83 (0.59–1.18) and 0.57 (0.40–0.83). Risk of stunting decreased with increasing education level. Compared with babies of mothers who belong to the apostolic sect, the following religions had lower risk of stunting: Roman Catholic, Protestant, Pentecostal, and no religion, OR (95% CI) 0.58 (0.39–0.88), 0.62 (0.48–0.79), 0.76 (0.62–0.93), and 0.09 (0.01–0.80), respectively. As expected, the risk of stunting decreased with increasing wealth quintiles. Finally, risk of stunting was higher for children whose mothers were HIV positive compared with children whose mothers were HIV negative, OR (95% CI) 1.32 (1.07–1.62).

From multivariate analysis and with reference to stunting, a girl child had significantly lower risk compared with a boy child, 0.77 (0.66–0.90). Having an obese mother had a lower risk compared to those with a thin mother, 0.68 (0.47–0.99). Children whose mothers belonged to the protestant or no religion had low risk of stunting when compared to children of mothers belonging to apostolic sect. Children whose mothers are in the richer and richest wealth quintile had lower risk of stunting compared with those whose mothers are in the poorest wealth quintile, 0.70 (0.50–0.98), 0.35 (0.22–0.57). Although of borderline significance, children whose mothers were HIV positive had a higher risk of stunting compared with those whose mothers were HIV negative, 1.23 (1.00–1.52). More details are presented in Table 3.

**TABLE 3:** Risk factors for with stunting, being underweight, and anemia among children enrolled in ZDHS 2015

Variable	Stunted	Stunted	Underweight	Underweight	Anemia	Anemia
	Unadjusted	Adjusted	Unadjusted	Adjusted	Unadjusted	Adjusted
	OR (95% CI)	OR (95% CI)	OR (95% CI)	OR (95% CI)	OR (95% CI)	OR (95% CI)
Baby gender						
Male	1	1	1	1	1	1
Female	0.77 (0.66–0.90)	0.77 (0.66–0.90)	0.80 (0.62–1.03)	0.79 (0.61–1.02)	0.87 (0.76–1.01)	0.87 (0.75–1.01)
Residence						
Urban	1	1	1	1	1	1
Rural	1.35 (1.13–1.61)	0.71 (0.50–1.02)	1.67 (1.22–2.30)	1.08 (0.60–1.96)	0.99 (0.84–1.17)	0.90 (0.65–1.23)
Mother's nutritional status						
Thin (BMI <18.5)	1	1	1	1	1	1
Normal (BMI 18.5–24.9)	0.83 (0.59–1.18)	0.86 (0.60–1.23)	0.43 (0.28–0.65)	0.44 (0.29–0.67)	1.01(0.71–1.43)	1.00 (0.70–1.43)
Overweight/obese (BMI ≥25)	0.57 (0.40–0.83)	0.68 (0.47–0.99)	0.18 (0.11–0.29)	0.21 (0.13–0.35)	0.79 (0.55–1.14)	0.81 (0.56–1.16)
Education level						
No education	1	1	1	1	1	
Primary	0.58 (0.31–1.07)	1.17 (0.98–1.40)	1.65 (0.50–5.43)	1.18 (0.89–1.57)	0.93(0.49–1.75)	1.00 (0.85–1.19)
Secondary	0.43(0.23–0.80)	‐	1.18 (0.36–3.85)		0.91 (0.48–1.71)	‐
Tertiary of higher	0.11 (0.05–0.26)	‐	0.38 (0.07–1.91)		0.64 (0.31–1.32)	‐
Religion						
Apostolic sect	1	1	1	1	1	1
Traditional	0.33 (0.08–1.35)	0.30 (0.06–1.43)	0.71 (0.16–3.14)	0.84 (0.19–3.81)	0.54 (0.19–1.53)	0.54 (0.18–1.64)
Roman catholic	0.58 (0.39–0.88)	0.74 (0.48–1.12)	0.65 (0.31–1.36)	0.87 (0.41–1.86)	0.93 (0.65–1.33)	0.95 (0.66–1.36)
Protestant	0.62 (0.48–0.79)	0.75 (0.58–0.97)	0.69 (0.47–1.03)	0.85 (0.56–1.29)	0.79 (0.62–1.00)	0.80 (0.62–1.02)
Pentecostal	0.76 (0.62–0.93)	0.92 (0.74–1.14)	0.76 (0.53–1.09)	0.97 (0.67–1.39)	1.09 (0.90–1.32)	1.12 (0.91–1.38)
Other Christian	1.10 (0.79–1.51)	1.18 (0.85–1.63)	1.03 (0.63–1.67)	1.10 (0.66–1.81)	0.92 (0.67–1.26)	0.93 (0.67–1.28)
Muslim	1.58 (0.45–5.51)	1.50 (0.41–5.54)	1.59 (0.32–7.94)	1.93 (0.40–9.26)	0.69 (0.18–2.70)	0.66 (0.17–2.64)
None	0.09 (0.01–0.80)	0.93 (0.68–1.28)	1.24 (0.78–1.98)	1.20 (0.75–1.92)	0.99 (0.73–1.35)	0.97 (0.71–1.32)8.09
Other	1.00 (0.73–1.36)	0.08 (0.01–0.70)	‐	‐	9.06 (0.93–88.02)	(0.82–80.12)
Wealth quintile						
Poorest	1	1	1	1	1	1
Poorer	0.81 (0.65–1.01)	0.85 (0.69–1.06)	0.88 (0.63–1.23)	0.95 (0.68–1.34)	0.70 (0.57–0.87)	0.72 (0.57–0.89)
Middle	0.73 (0.58–0.92)	0.80 (0.63–1.01)	0.69 (0.48–1.00)	0.78 (0.53–1.13)	0.87 (0.69–1.08)	0.92 (0.73–1.16)
Richer	0.75 (0.60–0.93)	0.70 (0.50–0.98)	0.62 (0.43–0.90)	0.85 (0.50–1.43)	0.90 (0.73–1.11)	0.88 (0.65–1.20)
Richest	0.38 (0.29–0.50)	0.35 (0.22–0.57)	0.38 (0.23–0.62)	0.65 (0.29–1.46)	0.77 (0.61–0.98)	0.76 (0.51–1.14)
HIV status						
Negative	1	1	1	1	1	1
Positive	1.32 (1.07–1.62)	1.23 (1.00–1.52)	1.69 (1.24–2.30)	1.49 (1.08–2.05)	1.20 (0.98–1.47)	1.17 (0.95–1.43)

#### 3.2.2 Being underweight

From univariate analysis and with reference to being underweight, although it was of the borderline significance, gender was associated with a risk of being underweight. Children residing in rural area had a higher underweight risk as compared to their urban counterparts. Mothers’ BMI was significantly associated with a risk of being underweight, with children of thin mothers being affected the most. The risk of being underweight decreased with increasing wealth quintile. Maternal positive HIV status is associated with an increased risk of the child being underweight, OR (95% CI) 1.69 (1.24–2.30). From multivariate analysis, maternal body mass index (bmi), being thin, and positive HIV status remained the only significant factors associated with the risk of being underweight. More details are presented in Table 3.

#### 3.2.3 Anemia

**FIGURE 1:**
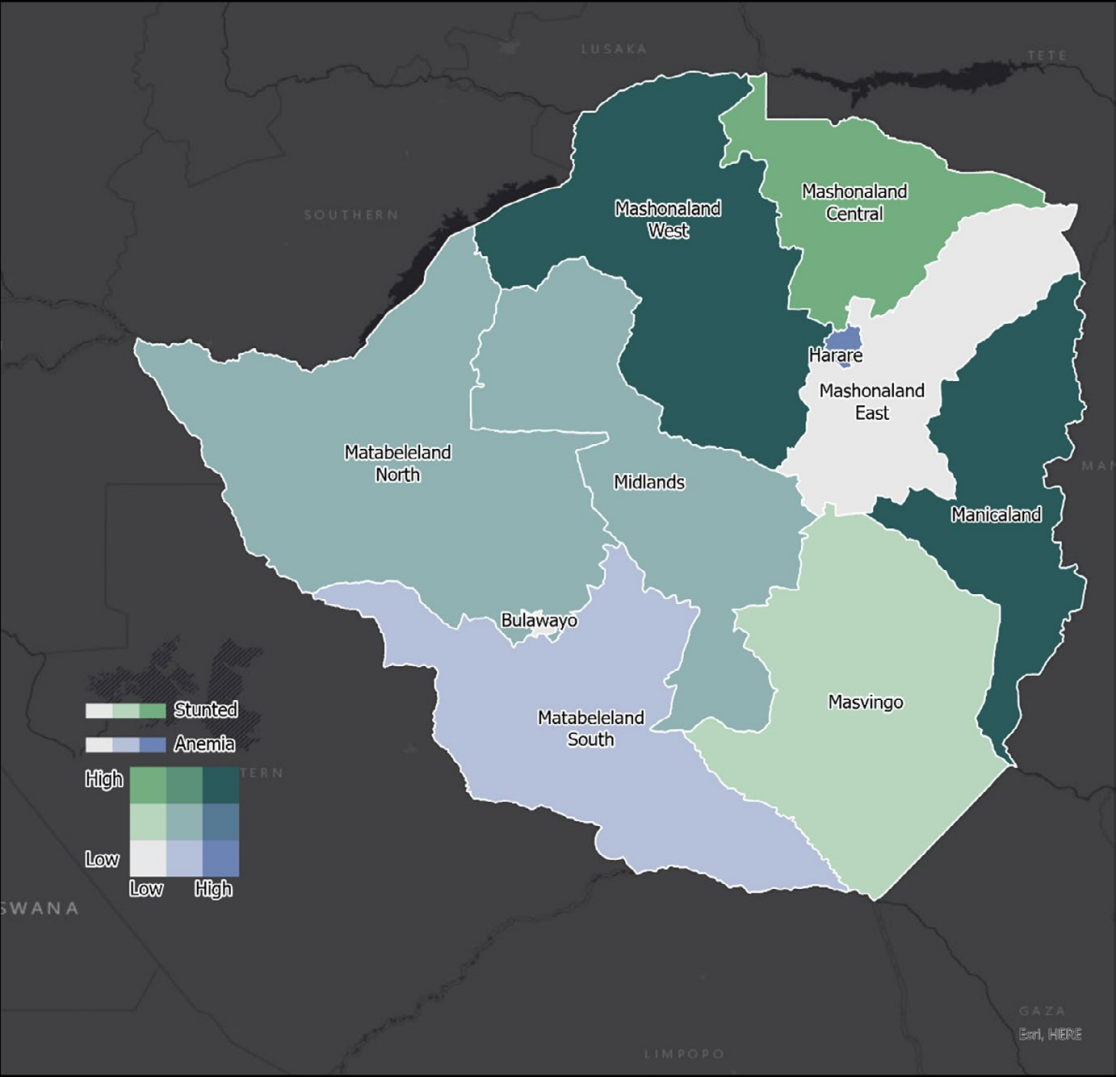
Provincial distribution of stunting and anemia in children

**FIGURE 2:**
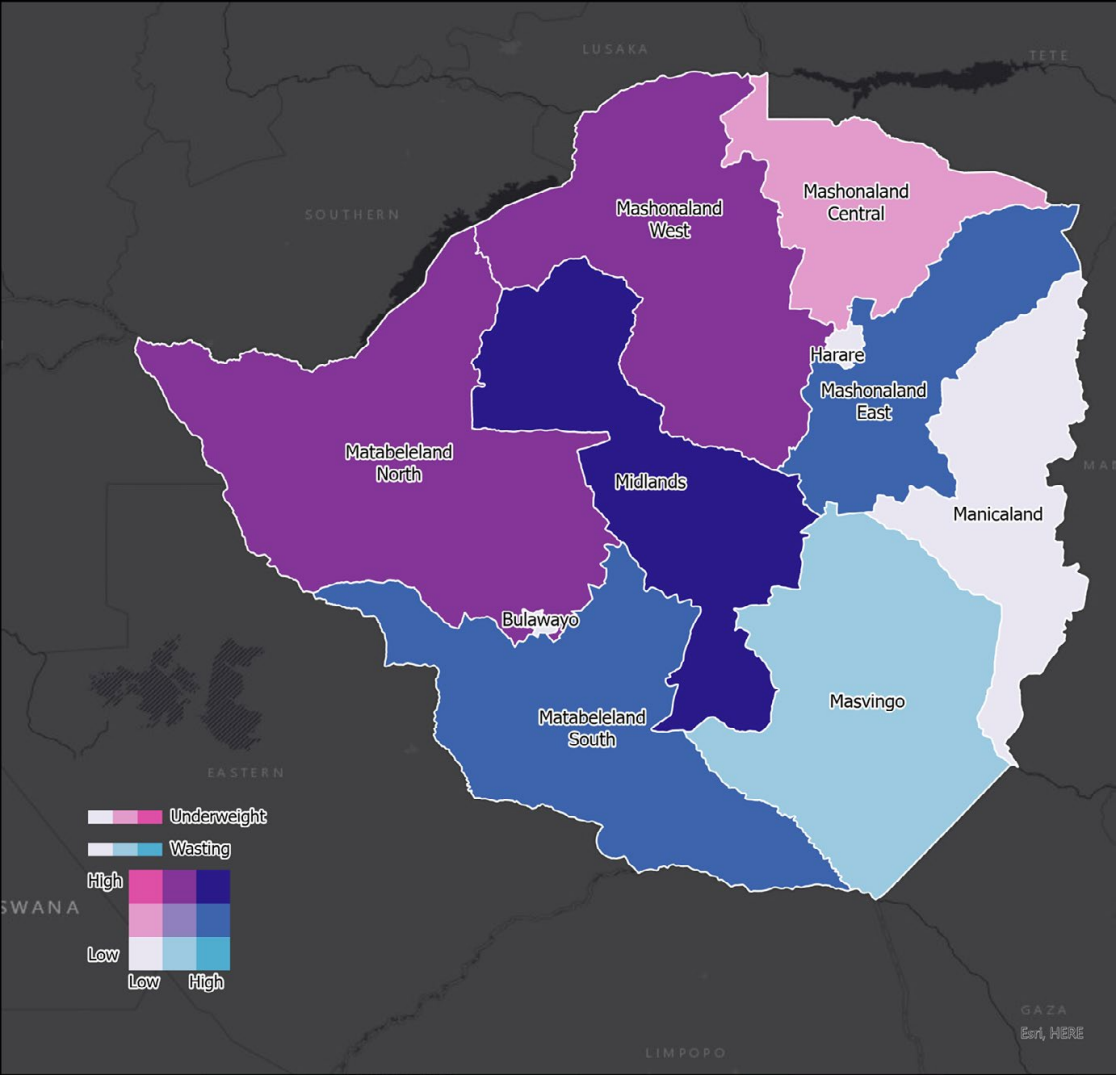
Provincial distribution of wasting and underweight status in children

In univariate analysis and with reference to anemia, female children had a lower risk of anemia compared with their male counterparts, although this is of borderline statistically significant. Wealth index was associated with risk of anemia with the richest having a significant lower risk. HIV positive mother's children had a borderline significantly higher risk of anemia. In multivariate analysis, female gender remained a borderline significant factor associated with risk of anemia. Wealth quintile direction of association remained the same, but with only a significant association recognized when comparing the poorer to the poorest. More details are presented in Table 3. The bivariate maps presented below show that Harare province has low stunting but high anemia whereas Mashonaland West and Manicaland have high prevalence of both anemia and stunting (Figure 1). Midlands province showed both high underweight and high wasting levels, while Masvingo had low underweight but high wasting. (Figure 2).

## 4 DISCUSSION

Unfortunately, the HIV status may affect many socioeconomic indices and directly or indirectly cause challenges on the offspring. Science must continue to investigate the direct and indirect impact of the HIV epidemic on women and their families; therefore, research must continue. In this study, we aimed to explore the association between maternal HIV status and childhood malnutrition, regardless of the HIV status of the children. Elucidating these associations is critical for devising interventions aimed at minimizing the impact of the HIV epidemic on family well‐being.

Previous studies have demonstrated the relationship between maternal HIV status and nutritional disorders. In one of these studies, the prevalence of stunting, underweight, and wasting was 14.2%, 8.0%, and 3.9%, respectively (Muhangi et al., 2013). In another study in Uganda, which shares similar population characteristics with Zimbabwe, there was evidence suggesting an association between maternal HIV infection and being underweight (aOR 2.32, 95% CI 1.32–4.09, *p* = .006). We noted similar findings in our study (aOR 1.49, 95% CI 1.08–2.05). In a prospective cohort study in Tanzania, there was a higher prevalence of stunting and underweight among HIV‐exposed infants who received less complementary feeding and had a less diverse diet (Kamenju et al., 2017). The findings add to the pool of evidence regarding maternal HIV status and undernutrition. In a secondary analysis of Demographic and Health Survey (DHS) data from 18 countries in sub‐Saharan Africa, HIV‐exposed children were significantly more likely to be stunted, underweight, or wasted compared with their unexposed counterparts, regardless of the exposed infant's HIV status (Magadi, 2011).

We noted a higher prevalence of infant anemia among HIV‐exposed infants in this study. Similarly, in a comparative cross‐sectional study, infantile iron‐deficiency anemia was associated with maternal HIV infection (aOR 2.54, 95% CI 1.65–1.39) (Magadi, 2011). However, these findings, from a population‐based survey similar to ours, may lack appropriate control for other confounding variables and effect modifiers. Indeed, other factors such as low‐income, residence, educational status of mother, age of the mother, and family size, which may also be associated with maternal HIV infection and infant undernutrition, have also been noted to be associated with infantile anemia (Feleke, 2016; Muhangi et al., 2013).

Several years into HIV Care and Treatment (HCT) programs, the results of this study still suggest a high prevalence of underweight, stunting, wasting, and infantile anemia among children whose mothers are HIV positive. Integration of feeding programmes into maternal care programs has not happened adequately, and possibly, not much has been done yet to deal with the maternal factors that contribute to undernutrition among HIV‐exposed infants and children. Young mothers, who are socio‐economically disadvantaged, are still at a much higher risk of HIV infection. The focus of public health must be to substantially reduce their risk of HIV acquisition, but also to adequately address factors that adversely affect their socioeconomic well‐being, which indirectly impact their children. On the other hand, there is a need for concerted efforts to integrate infant programs into maternal HCT programs, to improve the infants’ nutritional status, and improve their outcomes, including school performance and reduction in morbidity and mortality associated with nutritional disorders.

Since our geospatial maps show that stunting, anemia, underweight, and wasting are prevalent in the country, we recommend that the Zimbabwe Ministry of Health and Child Care implement a robust national wide improved infant and young child feeding (IYCF) scheme to enhance the overall nutritional status of children in the country. Interventions such as what we propose will go a long way in addressing this challenge.

The main limitation of this study is that the ZHDS was a cross‐sectional survey and collected only one biomarker, the HIV status. Therefore, some associations cannot be stated with certainty, and further studies are needed. It is also a limitation that we were not able to analyze the effects of children's HIV status. This was because very few children were HIV positive due to the successful PMTCT programs in Zimbabwe. This negligible number of children who are HIV‐positive defeats attempts to make any sound statistical disaggregation. Another limitation is that the survey did not collect Viral load Suppression and CD4+data. Since this was secondary data analysis, the chosen variables were entirely dependent on the number and nature of variables that were collected in the ZDHS.

## 5 CONCLUSIONS

Despite advances in HIV Care, Treatment, and Prevention, women of reproductive age continue to suffer the greatest burden of HIV in Zimbabwe. Living with HIV and AIDS still places a strain on maternal well‐being. Unfortunately, the HIV status may affect many socioeconomic indices, and the prevalence of infantile underweight, stunting, and wasting is high among HIV‐exposed infants. This study's findings call for implementation of a robust national wide improved infant and young child feeding (IYCF) scheme to enhance the overall nutritional status of children in the country including those from HIV‐positive mothers.

## AUTHOR CONTRIBUTIONS

**Godfrey N. Musuka:** Conceptualization (equal); Formal analysis (equal); Methodology (equal); Supervision (equal); Writing‐original draft (equal); Writing‐review & editing (equal). **Tafadzwa Dzinamarira:** Methodology (equal); Writing‐original draft (equal); Writing‐review & editing (equal). **Diego F Cuadros:** Formal analysis (equal); Methodology (equal); Writing‐review & editing (equal). **Grant Murewanhema:** Methodology (equal); Validation (equal); Writing‐review & editing (equal). **Innocent Chingombe:** Conceptualization (equal); Formal analysis (equal); Supervision (equal); Writing‐original draft (equal); Writing‐review & editing (equal). **Felicia Takavarasha:** Data curation (equal); Formal analysis (equal); Methodology (equal); Writing‐review & editing (equal). **Helena Herrera:** Conceptualization (equal); Validation (equal); Writing‐original draft (equal); Writing‐review & editing (equal). **Munyaradzi Mapingure:** Conceptualization (equal); Data curation (equal); Formal analysis (equal); Methodology (equal); Validation (equal); Writing‐original draft (equal); Writing‐review & editing (equal).

## Data Availability

The data that support the findings of this study are available from the Demographic and Health Surveys (http://www.measuredhs.com) but restrictions apply to the availability of these data, which were used under license for the current study, and so are not publicly available.
